# New York Academy of Medicine

**Published:** 1880-04

**Authors:** 


### NEW YORK ACADEMY OF MEDICINE.

Dr. P. S. Conner made some extended remarks of "cerebral localization from a surgical stand point." The subject was beautifully illustrated with large crayon and colored drawings, charts, etc.; and drew out an interesting discussion.

Dr. Ransohoff said that the subject, although a very great one, had been treated so exhaustively by his friend Prof. Conner, that the Academy would hardly find it interesting to listen to what little he had to say about it. There was, however, one point of interest which strikes the student in this particular department: Although the operation of opening the skull had existed since prehistoric times, no idea of localization of the motor and sensory centers seemed ever to have been excited.

As late as 1831 a French soldier received an injury on the left side of the cranium. There was paralysis of the opposite side following jactitation of the extremities. Examination revealed a depressed fracture with a spiculum of bone penetrating the brain. On moving the spiculum jactitation of the extremities was greatly increased, but all this did not provoke any ideas of cerebral localization. Other cases of injury of a similar character followed; symptoms of a like nature were noticed, but the fact of localization slept on unused and unknown until within the last few years physiological experiments for this purpose were performed upon animals. He considered deductions made from pathological of much more importance than those made from physiological research. The brain of the animal was not homologous to that of man. Removal of brain substance might not permanently destroy the function over which it presides in animals but it certainly would do so in man. In the former the centre, after removal, might be imperfectly reproduced, but it could not be so in man.

There had been a time in France when the operation of trephining was discarded for about a century, too much danger being attached to it.

Velpeau, however, did not consider it hazardous. The speaker considered that by relieving pressure and giving vent to collections of pus the operation should be performed, even though oftentimes it might only afford temporary relief. With antiseptic precautions it was considered much less objectionable. The great terror associated with the operation in Paris had been the occurrence of pyæmia; and when it was performed under different circumstances, with pure air and perfect cleanliness this did not follow.

In regard to the location at which to plant the crown of the trephine he agreed with the speaker who preceded him. Lucas Champoinniere had collected 173 cases of traumatic paralysis which occurred during the war of the rebellion, and in 139 of these the lesions had been found to exist in the space covered by the anterior half of the parietal bone. In fact, no well authenticated case of vertical paralysis had ever occurred the corresponding lesion of which might not be found in this psycho-motor area. The speaker reported two cases illustrating the subject under discussion.

He had been called to see a case with Dr. Zinke where the man had received an injury on the right side of his head, followed by jactitation of the upper extremity and muscles of the face on the opposite side of the body. This man recovered, but could not raise his left arm, and had aphasia. In a short time, however, he grew worse and died. At the post mortem examination a depressed fracture of the internal table of the anterior portion of the right parietal bone was found under which was an accumulation of pus. The external table was not affected by the injury.

The speaker regreted very much that the operation of trephining had not been performed at the point of injury in this case.

The second case was that of a young lady who had received an injury on the top of her head, from a falling body, while on the street. A depressed fracture of the skull with loss of consciousness was the result.

In six hours afterward when he first saw the case, consciousness had returned, but there was jactitation of the lower extremities which increased in the night, occurring about every fifteen minutes. Inflammation and serious symptoms coming on, he removed the portion of depressed bone, which was about three-fourths of an inch square, and traversed through its centre by the saggital suture. The fragment was passed around for inspection. The spasms of the lower extremities disappeared at once and forever, and she made an excellent recovery.

The point of pressure in this case, he said, was the paracentral lobule or the part surmounting the upper end of the fissure of Rolando.

So far as trephining for epilepsy was concerned, he thought that in the majority of cases the operation would do no good. If the disease was of traumatic origin most surgeons would agree that very little was to be expected from an operation. In cases where the brain had been subject to long pressure, even if the surgeon was so fortunate as to be able to remove the offending element with the trephine, the brain tissue would not speedily return to its normal condition, and the so-called epileptic habit would continue. Paul Eve, of Nashville, had at one time removed a button of bone from a point immediately over the lateral sinus, and the epilepsy, for the relief of which the operation was performed, disappeared at once; but it afterwards returned and was as troublesome as ever.

Six years ago he had seen a man come into the hospital and immediately afterwards have a slight epileptoid attack in which he became listless, but not unconscious. Six or eight hours afterwards he had a violent epileptic fit, upon which the speaker examined him critically, and found that he had been trephined for epilepsy following injury of the head, and that the disease had been releived but for a short time only.

Dr. Whitaker said that there were other aspects to this subject, besides the surgical, of equal interest and promise.

The first suggestions which led to the study of cerebral localization had been made by a Frenchman, Serres, in 1823. In 1861 the subject was studied by Dax, Bouillaud and Broca, who gave us the language centre, and ten years afterwards Fritsch and Hitzig first demonstrated psychomotor centres in the cortex of the brain. Then, among many others, came Ferrier, who had experimented so extensively on the lower animals, and found that the psycho-motor centres of the cat, the dog and the monkey were not exactly the same.

The localized areas in the brain of man could, therefore, be only assumed from analogy in the monkey. But clinical and pathological evidence lent confirmatory proof of the existence of motor-centres in the cerebral cortex.

After Ferrier came Nothnagel, Carville, Duret, the Italian physiologists Sussana, and Schiff, and Brown-Sequard, who was devoting the last half of his life to refute the evidence collected in the first half.

Dupuy cauterized the brain of animals and destroying the convolutions, found that, after a month, irritation of the eschar caused the muscles to respond in the same manner as if the brain had been left intact, and Schiff, of Florence, as the result of his studies, had utterly disclaimed the localization of psycho-motor centres in the cerebral cortex. The opponents of such localizations did not dispute the results of the experiments, that is, the movements of muscles in response to excitation of the brain, but explained them by a reflex action upon the ganglia at the base, which have always been undisputed centres of motion.

Nor did Ferrier deny the results of Dupuy, that is, of persistence of response after cauterization of the cerebral cortex, but explained it by the well accepted doctrine that acts which at first require volition, the action hence of the cerebral cortex, becomes subsequently automatic, and are then presided over by the basal ganglia.

The speaker believed that in the present state of the question, the great bulk of evidence is in proof of the existence of psycho-motor centres in the brain. When limited movements resulted from limited excitation of the brain movements as definite as the sounds elicited by striking definite keys upon the piano-forte, there would hardly seem room for doubt. Thus, the psycho-motor zone had been limited to the region about the fissure of Rolando, the intellectual zone was in front of it, the sensory zone is behind it, in the parietal and post-parietal regions and the great mass behind this region, the mass which occupies the posterior fossa of the skull was devoted to general government, coordination, etc. These points seemed to be settled; none so clearly in man, however, as the localization of the psycho-motor zone, and here the contributions of Hughlings Jackson on epilepsy had contributed the main evidence.

It was not to be expected that exact centres could be determined as yet, that is, could be precisely circumscribed, for the reason, that the brain of man is still under development. It was more true to say that centres are being differentiated and set apart for special functions, than that they have been already so perfected and separated from each other. This was a point which had been strangely overlooked in the study of this subject, even by individuals whose education is modern enough to include the theory of evolution.

He objected to the term "epileptic habit" being applied to the persistent tendency to the recurrence of the disease. He thought that in this condition a part of the cause that produced the epilepsy remains. Hughlings Jackson had long ago explained these phenomena by pointing out the nature of the discharging lesions of the brain, and he considered the term, "epileptic habit," one of those unfortunate survivals from a time when diseases were looked upon as other things than lesions of structure. So the old obstetricians spoke of a "habit of abortion," which, when we came to investigate, was found to depend for the most part upon syphilis, a maternal blood-poison that we could understand. In speaking of epilepsy persisting after removal of the cause, it would be better to recognize that all the cause has not been removed, but that the nerve-tissue or other seat of injury of disease is still subject to irritation. A part only, and not all the cause has been removed. In thinking in this way we would keep to solid ground and away from any speculation.

Dr. Vance remarked that the discussion which he had just listened to with so much interest had revived old associations, and it seemed strange that a student of James R.

Wood should hear it said that trephining was out of vogue. He had seen that operator go down upon the brain repeatedly and bring out pus. The operation of scraping an aperture in the skull for the relief of epilepsy was more than antique, and trephining was destined to come into use again for the cure of the same disease.

A simple opening of the skull without other treatment would not cure epilepsy. He had seen a case in which a blow on the head had caused momentary loss of consciousness.

Some time after spasms of the right hand occurred. The man was a book-keeper and the spasms became so violent that he actually drove the pen into his face. Seeking medical advice he was told by one physician that it was only nervous, and by another that it might be the begining of epilepsy. The patient went to New York and consulted the speaker, who trephined a little anterior to the point marking the upper end of the fissure of Rolando. The attacks grew fewer and fewer and he finally recovered perfectly and was at present in Washington, a well man.

In 1869, at the Bellevue Hospital Medical College, the first course of lectures on nervous diseases in America was delivered by Wm. A. Hammond, and the speaker being his assistant used to prepare the animals for experiment. One day by accident he applied Faradic electricity to a convolution of the brain of a dog while under the anæsthetic, and the animal wagged his tail. He regretted that he had failed to utilize the accidental circumstance and so allow the laurels of demonstrating cerebral localization to pass to others.

In answer to a question he stated that the button of bone removed by the trephine in his case was rough and presented a slight spicular exostosis on its under surface. It resembled the conditions he had noticed in other cases of the same kind.

He was of the opinion that much good could be accomplished in using the trephine for epilepsy. Professor Van Buren and Stephen Smith were decidedly in favor of the method. There were other features, however, connected with the treatment which should not be overlooked. After removing the cause the epileptic habit should be controlled. Dr. Billings, of Washington, a graduate of the Ohio Medical College, collected and arranged in an inaugural thesis over sixty cases of epilepsy treated in this way of which more than forty were returned as cured.

Dr. Reamy doubted the propriety of trephining very often for epilepsy, though it was known that some cases of that disease of not very long standing, had been cured by removal of depressed *spicula* of bone. He did not think that epilepsy was absolutely incurable, or that the so-called habit could not be changed; for he had observed two idiopathic cases, one of six, and the other of three years standing, which had recovered under the administration of the bromides of lime, soda, and potassa given alternately and continued with intermissions for several years.

Dr. Conner in answer to a question, stated that the old method of scraping an opening in the skull for the re ief of epilepsy was still in use among some of the tribes of Africa.

As to trephining for epilepsy, according to his own personal observation, it had resulted in death in nearly every case. He believed that there was some good in the operation though in many cases it was unwarrantable. If it would produce relief, even without curing the disease, he considered the operation justifiable.

